# Characterizing lysinoalanine crosslinks in food systems: Discovery of a diagnostic ion in model peptides using MALDI mass spectrometry

**DOI:** 10.1016/j.fochx.2023.100800

**Published:** 2023-07-23

**Authors:** Hannah McKerchar, Jolon M. Dyer, Juliet A. Gerrard, Evelyne Maes, Stefan Clerens, Renwick C.J. Dobson

**Affiliations:** a Biomolecular Interaction Centre, School of Biological Sciences, University of Canterbury, Christchurch 8140, New Zealand; b Riddet Institute, Based Massey University, Palmerston North 4442, New Zealand; c Proteins and Metabolites Team, AgResearch Lincoln Research Centre, Lincoln 7608, New Zealand; d Department of Biochemistry and Molecular Biology, Bio21 Molecular Science and Biotechnology Institute, University of Melbourne, Victoria 3010, Australia; e School of Biological Sciences and School of Chemical Sciences, University of Auckland, Auckland, New Zealand; f The New Zealand Institute for Plant and Food Research, Lincoln Research Centre, Lincoln 7608, New Zealand; g Department of Wine, Food and Molecular Biosciences, Lincoln University, Lincoln 7647, New Zealand

**Keywords:** Lysinoalanine, Protein–protein crosslinks, Food processing, Mass spectrometry

## Abstract

Formation of lysinoalanine protein–protein crosslinks during food processing adversely impacts nutritional value. However, mapping lysinoalanine directly in food is challenging. We characterized the fragmentation pattern of lysinoalanine crosslinks in synthetic peptide models over a range of pH and time treatments using mass spectrometry. A putative diagnostic ion resulting from the cleavage of the α-carbon and β-carbon of lysinoalanine is identified in MALDI MS/MS spectra. This represents the first step in mapping lysinoalanine in real food samples with higher precision than currently identifiable through standard or customized software. We then determined a correlated trend in the reduction of disulfide bonds and formation of lysinoalanine with increasing pH and time. Mapping lysinoalanine formation is critical to enhance our understanding of molecular processes impacting the nutritional value of foods, including notably in the development of protein alternatives that use alkaline treatment to extract protein isolates.

## Highlights

• Mapping lysinoalanine crosslinks in foods is challenging.
• Lysinoalanine formation increases at pH 12 and over 12 days.
• Diagnostic ion for lysinoalanine crosslink is identified.
• Identification and location of lysinoalanine crosslink using MALDI TOF/TOF.

## 1 Introduction

Protein-protein crosslinks form in food proteins during common processing and preparation conditions, including elevated temperatures, high pH and long storage times (Friedman et al., 1984, Lassé et al., 2015). The formation of such process-induced crosslinks profoundly influence the properties of foods (Gerrard, 2002, McKerchar et al., 2019). A case-in-point is the formation of the undesirable crosslink lysinoalanine, which is favored by high temperatures and alkaline conditions (Dyer et al., 2013, Friedman, 1999, Friedman et al., 1984). Lysinoalanine is present in a wide range of processed foods, including: wheat based products (Chinese noodles, pretzels and crackers) (Hasegawa et al., 1987, Rombouts et al., 2012), fish (Kume & Takehisa, 1984), chicken (Sternberg & Kim, 1977), frankfurter sausages (Sternberg & Kim, 1977), eggs (Hasegawa & Iwata, 1982), cheeses (halloumi and mozzarella) (Pellegrino et al., 2010), milk products (Fritsch and Klostermeyer, 1981, Sternberg and Kim, 1977), baby food, gelatine (Fujimoto & Roughley, 1984), whipping agents (Fritsch and Klostermeyer, 1981, Sternberg and Kim, 1977), infant formulas (D'Agostina et al., 2003, Pellegrino et al., 1996) and enteral nutrition formula (Boschin et al., 2003). The relative prevalence of lysinoalanine is problematic because 1) it reduces the nutritional value of food and 2) the safety implications for humans is unresolved (Cartus, 2017, Friedman, 1999).

**Fig. 1:**
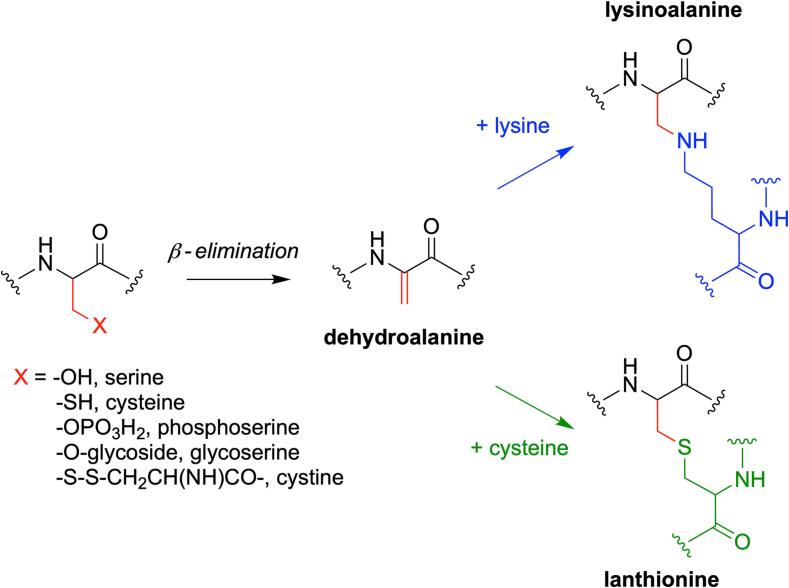
Formation of lysinoalanine and lanthionine crosslinks. The side chain (X) undergoes β-*elimination* to form the intermediate dehydroalanine. The double bond of dehydroalanine can react with either the amino group of lysine to form lysinoalanine, or the thiol of cysteine to form lanthionine.

Formation of lysinoalanine occurs in two steps (Fig. 1). In the first step, cysteine, serine, *O*-phosphorylserine, *O*-glycosylserine, or cystine undergo β-elimination to form the intermediate dehydroalanine (Friedman, 1999). In the second step, the double bond of the dehydroalanine intermediate can either react with the nucleophilic ε-amino group of lysine to form lysinoalanine, or react with the thiol of cysteine to form lanthionine (Friedman, 1999).

Lysinoalanine reduces the nutritional value of food in a number of ways, including: 1) by reducing the levels of the parent lysine residue, an essential amino acid; 2) by impairing the accessibility of proteolytic enzymes to the protein; and 3) by inhibiting metalloenzymes (Boschin et al., 2003, Friedman, 1999). Suggestions of toxicity have also been raised, though the mechanism is not well understood and effects are dependent on source, dose duration and species that consume lysinoalanine (Friedman, 2008).

Currently, only the total levels of lysinoalanine can be measured after protein hydrolysis and derivatization, which loses all locational information. To fully understand and therefore allow mitigation of the undesirable effects of lysinoalanine in food processing, it is critical to identify and locate and map these crosslinks within a protein. The problem in locating lysinoalanine is that food is chemically very complex, with a multitude of potential interactions and reactions between components. Crosslink formation occurs within single peptides/proteins and between two or more different peptides/proteins. Foods’ complexity necessitates a simpler model system to investigate the chemistry that takes place when lysinoalanine forms. By mapping where crosslinks occur in a protein, we can better understand the conformation of proteins when digested and how they might behave upon digestion. It also provides insight into how a proteins’ structure, after processing, influences the properties of the food.

Our approach to the problem is to engineer a model lysinoalanine crosslink between synthetic peptides, which enables us to focus on the chemistry and observe what happens when two peptides react. Characterizing the chemistry of the peptide model allows us to understand what happens in food, which would not otherwise be possible. Interrogating the crosslink model using mass spectrometry offers means of identifying a characteristic that can be used to automatically locate the crosslink in food. This approach has the benefit of avoiding extractability issues caused by crosslink aggregation and reducing complexities introduced by concurrent modifications (Lebendiker and Danieli, 2014, Maes et al., 2017). Optimizing the conditions for engineering a crosslink offers an attractive means of easily enriching crosslinked peptides compared to more dominant linear peptides. Tracking how formation trends change when conditions are varied gives insight into the relationship between the different crosslinks that can form.

Characterizing a peptide crosslink model has the added advantage of avoiding use of proteolytic enzymes. Digestion of crosslinked proteins differs from standard proteomic protocols with added challenges (Maes et al., 2017). The most common enzyme, trypsin, is ineffective on a crosslinked lysine, which occurs when lysinoalanine forms (Leitner et al., 2014). Chymotrypsin can cleave isolated proteins containing lysinoalanine (and the related crosslinks lanthionine, 3-methyl-lanthionine and 3-methyl-lysinoalanine) (Rombouts et al., 2016). Rombouts et al. (2016) successfully located the crosslinks locations using mass spectrometry on a protein-by-protein basis, with elements of manual calculation, in a simple system. However, in complex food samples the enormous number of variables and potential peptide combinations make proteolytic digestion and manual assessment unfeasible.

Automation is essential to identifying crosslinks amongst the thousands of spectra dominated by greater levels of linear, un-crosslinked, peptides in food samples. Defining an easy diagnostic for locating lysinoalanine within a protein is important as there is currently no automated means of detecting lysinoalanine in mass spectrometry data on a proteomic level. Typically, experimental mass spectra are compared to a database containing theoretical masses using computational algorithms that identify proteins and map modifications. However, without knowing where a crosslink forms in the protein sequence, or the resulting mass shift, as is the case with lysinoalanine, mapping the crosslink from the observed data is very challenging. During the database search, each single peptide must be combined with every other peptide to create the correct pair that forms a crosslink. As a result, the number of possible crosslinked peptides in the databases increases quadratically with the number of linear peptides, where (*n*^2^ + *n*)/2 possible pairs for *n* peptides are theoretically available for crosslinking (Rappsilber, 2011). Searching for crosslinks leads to a computational explosion that has hindered software development. Inroads into the problem have been made with custom-written software developed for specific crosslinks (but not lysinoalanine) by utilizing diagnostic ions or diagnostic fragmentation patterns of crosslinked peptides (Courcelles et al., 2017, Gao et al., 2006, Leitner et al., 2012, Maiolica et al., 2007, Riffle et al., 2016, Rinner et al., 2008, Yugandhar et al., 2020). Customized software has yet to be developed to automatically map lysinoalanine, this is partly due to the lack of understanding of the mechanisms of crosslink and the absence of a diagnostic ion.

Here, we identify a diagnostic fragmentation pattern using matrix assisted laser desorption ionization-time of flight/time of flight (MALDI-TOF/TOF) mass spectra, which could be used to customize software with algorithms that identify lysinoalanine when database searching. Customizing software enables large data sets to be analyzed automatically for lysinoalanine, negating the need for time consuming manual analysis previously used on a protein-by-protein basis. Our results are the first critical step in creating software that locates lysinoalanine in an automated way that will underpin future efforts to probe the effect of lysinoalanine on the structure of proteins and quality and safety of food in real food samples.

## 2 Materials and methods

### 2.1 Materials

Mass spectrometry grade water, trifluoroacetic acid (TFA), sodium hydroxide, dithiothreitol (DTT) and ammonium bicarbonate were purchased from Fisher Scientific (Sunnyvale, CA, USA). Mass spectrometry-grade acetonitrile (ACN) and formic acid (FA) were purchased from Fluka Analytical, by Merck (Kenilworth, NJ, USA). Hydrogen chloride was purchased from Univar (Downers Grove, IL, USA). Equipment used in MALDI-TOF mass spectrometry including Anchorchip target plate, a-cyano-4-hydroxy-cinnamic acid matrix and peptide calibrant standard II were all purchased from Bruker (Bremen, Germany).

### 2.2 Synthetic linear peptides

Three linear peptides, with the sequences LLSLR (49% pure), LLKLF (93% pure), and LKDECFR (95% pure) were supplied by Auspep Pty Ltd (Tullamarine, Victoria, Australia), or by Mimotopes (Clayton, Victoria, Australia). Modifications were made to the amino acid residues on the ends of the peptides by adding protecting groups to help make them more resistant to the harsh alkaline and heat treatment conducive to lysinoalanine formation (Ac-LLSLR-NH_2_, Ac-LLKLF-NH_2_, and Ac-LKDECFR-NO_2_).

### 2.3 Purification of peptides by high performance liquid chromatography

The synthetic linear peptides were purified by high performance liquid chromatography (HPLC) using an Ultimate 3000 HPLC system fitted with a Famos autosampler and a photodiode array detector (Dionex, Thermo Fisher, Sunnyvale, CA, USA). Peptides were dissolved in 0.1% TFA (2 mg/mL). Separation was achieved using a reverse phase Zorbax SB-C18 (205 × 4.6 mm, 5 µm particle size) column (Agilent Technologies, Santa Clara, CA, USA), where 50 µL of peptide was injected at a flow rate of 1 mL/min at 40 °C. Separation time was 22 min. The starting condition for each run was 75:25 mobile phase A (0.1% TFA)/ mobile phase B (90% ACN and 0.1% TFA). A linear gradient from 25 to 100% of mobile phase B was used over 22 min for peptide elution. At the end of each run the column was washed with 100% mobile phase B for 2 min, which was then reduced to 25% mobile phase B over 1 min, the column was then re-equilibrated with 25% mobile phase B for 10 min before injecting the next sample. The peptide elution was monitored by measuring the absorbance at wavelength 218 nm using a photodiode array detector (Ultimate 3000 PDA). Identification of the peaks relating to the peptides was guided by retention times provided by the manufacturer that used the same type of column. Three-dimensional data were collected and plotted using Chromeleon 7.2 Chromatography Data System software (Thermo Fisher, Sunnyvale, CA, USA). Eluted fractions correlating to the peptide peak were collected, pooled and aliquoted and stored at −80 °C until further use.

### 2.4 Application of stressors to peptides and sample preparation for mass spectrometry

Purified peptide samples were dried under vacuum (Savant speed-vac, SC-100) and re-solubilized 1:4 with sodium hydroxide (0.01 M) with pH of either 6, 8, 10, 11, or 12 as adjusted with hydrochloric acid. Concentrations were determined using a Direct Detect spectrometer (Merck) according to manufacturer's instructions. Single peptide solutions and mixtures of peptide solutions were normalized to the same concentration before being combined in a 150 µL Reacti-Vial (Thermo Fisher Scientific Inc., Sunnyvale, CA, USA) and sealed tightly to prevent evaporation. Peptides were heated in an oven at 70 °C or left at room temperature for either 3 h, 1, 2, 4, 6, or 12 days. Application of heat was stopped by placing the Reacti-Vial on ice for two minutes before being placed at ambient temperature. Alkaline conditions were neutralized with the addition of 0.01 M HCl until the pH measured pH 7. Salts were removed from the peptide mixtures with Pierce™ C18 Stage-tips, 200 µL (Thermo Fisher Scientific Inc., Sunnyvale, CA, USA). The tip was conditioned with 80% ACN, 1% FA (20 µL), re-equilibrated with 1% FA (20 µL), before the peptide mixture was loaded (20 µL) and washed with 1% FA (20 µL). Samples were eluted from the tip in a solution of 50% ACN, 1% FA (10 µL).

Before MALDI-MS analysis, FA was removed by evaporation by drying under vacuum (Savant speed-vac, SC-100). Samples were then resuspended in the same volume of 0.1% TFA acid prior to evaporation. Each sample was prepared in duplicate.

### 2.5 Confirming sequence of linear peptides by off-line nano-electrospray ionization ion trap mass spectrometry and assessing concurrent modifications

Direct injection off-line nano-electrospray ionization (ESI) on an amaZon Speed ETD ion trap mass spectrometer (Bruker, Bremen, Germany) was used to confirm the sequence of purified linear peptides. Modifications that occurred on the purified linear peptides as a result of applying high temperature for varying lengths of time at pH 12 were then assessed.

Samples of an untreated peptide solution was compared with a peptide solution, having the same sequence and concentration but heated at 70 °C for 3 h at pH 12 as described (section 2.4). The peptide solutions were loaded into the mass spectrometer using a 1 mm capillary tip from EconoTips (New Objective, Scientific Instrument Services Inc, New Jersey, USA). Ionization was carried out in positive mode; a voltage of 800 V was applied to the capillary, the ion charge control was set to 100,000 ions at an accumulation time of 40 ms and a rolling average of 5 was set. Nebuliser gas nitrogen was pressurized to 20 kPa that was added at a dry gas rate of 3.0 L/min and a dry temperature of 150 °C. Mass spectra were collected for a minimum of 2 min. Data were acquired in a range of *m*/*z* 100–2000. Collision induced dissociation (CID) was used to fragment precursor ions. Collision energy was increased over time from zero volts until no precursor ion remained, the *m*/*z* scale was adjusted to the *m*/*z* of the parent analyte. Data were analyzed by manual annotation through *de novo* sequencing using flexAnalysis 3.4 (Bruker Daltonics, Germany).

### 2.6 Matrix assisted laser desorption ionization - time of flight analysis

Samples were spotted using the parafilm wax film preparation method for matrix assisted laser desorption ionization - time of flight (MALDI-TOF) analysis (Wang et al., 2008). One microlitre of sample was spotted on parafilm wax film, 1 µL of freshly prepared matrix solution was applied on top and mixed with a pipette. The freshly prepared matrix solution contained acetone (100 µL) saturated with α-cyano-4-hydroxycinnamic acid matrix that had been sonicated and diluted 10 times in a solution of ethanol (600 µL), acetone (300 µL) and 0.1% TFA (100 µL). One microlitre of the sample/matrix solution was directly spotted onto an AnchorChip plate and co-crystallized by evaporating to dryness at ambient temperature.

The plate was loaded in to an Ultraflex III MALDI-TOF mass spectrometer (Bruker) and analysis carried out in positive reflectron mode. A nitrogen laser operating at 355 nm was shot randomly at the sample and a total of 1500 shots were summed. Data were acquired using flexControl 3.0 software (Bruker) over a *m*/*z* range 700–3000. The peptide calibration standard (Bruker) containing nine standard peptides, was prepared according to the manufacturer’s instructions and spotted in the same way as the samples. The mass spectrometer was calibrated with all nine standard peptides that had a *m*/*z* range of 757.39–3147.47, each standard peptide was within 10 ppm of the reference mass.

MALDI time-of-flight/time-of-flight (MALDI-TOF/TOF) was used to fragment precursor ions. LIFT™ was used with time-of-flight/time-of-flight (LIFT-TOF/TOF) to elevate the potential of the collision cell above that of the ion source. Argon gas was added to enhance fragmentation. Precursor ions were manually selected from the MALDI TOF mass list.

Data were processed in flexAnalysis 3.4 (Bruker Daltonics, Germany). The protein mass fingerprinting flex analysis mass spectrometry (PMS.FAMS) method, which uses the sophisticated numerical annotation procedure (SNAP) algorithm to pick peaks from isotope patterns, was used to process MS/MS spectra. Manual *de novo* sequencing and annotation was then undertaken. The MS error tolerance was set to 0.2 Da and the MS/MS error tolerance was set to 0.8 Da.

## 3 Results and discussion

To reduce the complexity of mapping lysinoalanine in a food, we engineered model synthetic disulfide, lanthionine and lysinoalanine crosslinks from linear peptides and characterized their fragmentation spectra *de novo*. We designed the peptide sequences such that they ionize well, have low steric hindrance around the residues that take part in lysinoalanine formation, and to minimize side reactions. The primary sequences of the linear peptides were LLSLR, LLKLF and LKDECFR. Modifications to the termini were included to increase resistant to harsh alkaline and heat treatment used to optimize crosslink formation. The influence of pH and exposure time to heat expected to induce the formation of lysinoalanine was defined.

### 3.1 Modifications and an additional site for crosslinking occur with heating at pH 12

Before applying any stressors to the synthetic linear peptides by heating or changing the pH environment, we checked their purity and sequences (LLSLR, LLKLF and LKDECFR) using off-line direct injection nano-electrospray on an ion trap mass spectrometer. The observed masses were compared to the theoretical monoisotopic mass of each peptide as calculated *in silico* using the Peptide Mass Finder tool (Wilkins et al., 1997). Theoretical masses were within Δ0.01 Da of the observed mass at the apex of each peptide peak in unprocessed MS spectra. Sequences were confirmed from the MS/MS spectra.

**Fig. 2:**
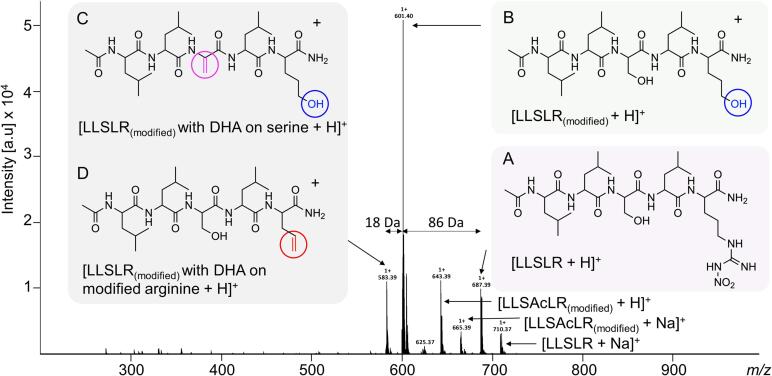
MS spectrum of LLSLR following heating at pH 12. Off-line nano-electrospray direct injection ESI ion trap MS spectra (*m/z* range of 200–1000) of LLSLR suspended in 0.01 M NaOH at pH 12 heated for 3 h at 75 °C. The structure of ‘unmodified’ linear peptide LLSLR at *m/z* 687.39 is labelled A ( box), the structure of ‘modified’ arginine peptide (LLSLR_(modified)_) with hydroxyl modification to arginine shown in  circle at *m/z* 601.40 is labelled B ( box), the structure of LLSLR_(modified)_ with dehydroalanine (DHA) formed at serine shown in  at *m/z* 583.39 is labelled C and the structure of LLSLR_(modified)_ with DHA formed at the modified arginine shown in  at *m/z* 583.39 is labelled D (both in the  box). (For interpretation of the references to colour in this figure legend, the reader is referred to the web version of this article.)

Following confirmation of the sequence, we determined whether modifications occur in the peptides upon heating at pH 12. Formation of lysinoalanine in soybeans is known to occur in pH conditions up to pH 12.5 and heating at 75 °C for 3 h (Friedman et al., 1984). Guided by these findings, we determined whether modifications occur on the linear peptides after heating at pH 12 at 70 °C for 3 hr. Given the severe conditions being applied to the linear peptides, modifications other than the formation lysinoalanine or crosslinks are likely. Any concurrent modification, in addition to lysinoalanine, will cause a mass shift that needs to be considered when determining for the mass shift due to the formation of the crosslink. Subjecting the linear peptide LLSLR to heat at 70 °C at pH 12 for 3 hrs resulted in a series of modifications, although the linear native protected LLSLR peptide could still be detected at *m*/*z* 687.39 (and with Na^+^ adducts at *m*/*z* 710.37) following treatment (labelled A. purple box, Fig. 2), but it was a minor species indicating that the peptide was indeed modified. In contrast, the two other linear peptides LLKLF and LKDECFR did not form concurrent modifications following treatment.

The first modification to linear peptide LLSLR was observed at *m*/*z* 601.40 (an 86 Da mass shift), which we attribute to modification of the C-terminal arginine residue. CH_2_N_4_O_2_ is lost from the NO_2_ protected side-chain and replaced with a δ-OH on the arginine (labelled B,  box, with δ-OH in blue, Fig. 2). The modification occurred despite a nitrile (NO_2_) being added during synthesis in an effort to protect the arginine side-chain to the harsh conditions. We refer to this treatment-induced modification as ‘modified’ arginine and the peptide as LLSLR_(modified)_. A related but minor modification of arginine was also observed at *m*/*z* 643.39 (and with Na+ adducts at *m*/*z* 665.39), which we attribute to an additional acetylation of serine, LLSAcLR_(modified)_ (structure not shown).

**Supplementary figure 1:**
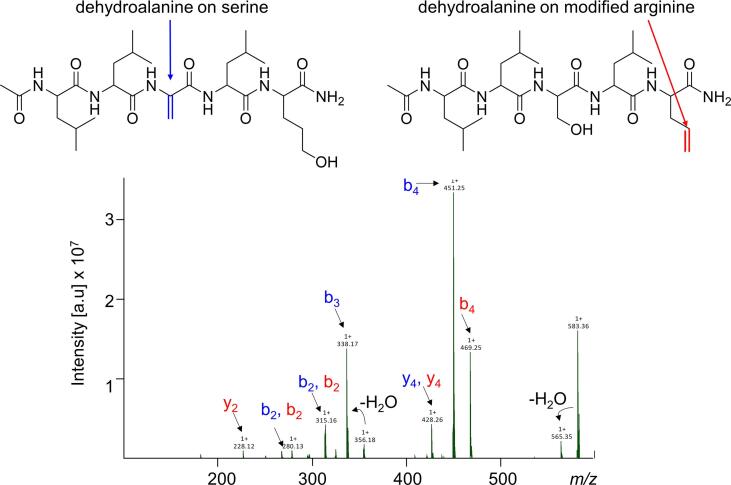


Two additional modifications were attributed to the peak a *m*/*z* 583.39; the formation of dehydroalanine (DHA) either on the serine or the arginine (labelled C and D,  box, Fig. 2)—both of which form from the LLSLR_(modified)_ peptide (labelled B,  box, Fig. 2). The treatment-induced modification at arginine introduces a second hydroxyl into the peptide (labelled B,  box in Fig. 2), in addition to the already present hydroxyl of serine. The alkaline conditions used in this experiment induced *β*-*elimination* of the hydroxyl moieties to form the DHA intermediate of lysinoalanine and lanthionine (Fig. 1). Dehydroalanine can form from either the hydroxyl at serine, as shown in magenta in the structure labelled C in Fig. 2, or at the modified arginine, as shown in the  circle in the structure labelled D in Fig. 2. Elimination of hydroxyl at either site has a Δ18 Da shift from the ‘modified’ peptide (labelled B,  box in Fig. 2) and each resulting peptide has the same mass, *m*/*z* 583.39 (C and D in  box in Fig. 2). The MS/MS spectrum of *m*/*z* 583.39 (LLSLR_(modified)_) (Fig. S1) contains fragments of each species, one with the DHA on the serine (C,  box in Fig. 2) and a second species with the DHA on at the modified arginine (D,  box in Fig. 2). A third species, with two DHA modifications at both the modified arginine and the serine (*m*/*z* 565.37) is detectable at low intensity in the MS spectrum (not highlighted in Fig. 2).

In summary, by subjecting linear peptides to heat and time stressors, we found a treatment-induced modification that introduced a second site where lysinoalanine can form on the linear peptide LLSLR. This concurrent modification occurred on the C-terminal arginine residue of the LLSLR peptide. This relatively simple modification to the arginine residue increases the number of places lysinoalanine crosslinks can form. The repercussions of one modification in this unsophisticated model, exemplifies the complexity concurrent modifications cause when mapping process-induced crosslinks and how the number of possible peptide combinations in a crosslink can escalate. Scaling up to a food sample requires accounting all of modifications that could occur, all the subsequent flow-on modifications and then accounting for all the possible modified peptides that could potentially crosslink. The task is exceptionally complex and is a major challenge in locating process-induced crosslinks on a proteomic level. The modification to our model peptide also demonstrates the need to closely examine the MS/MS spectrum to determine where the modification is located, which is not possible from the MS spectrum. Nonetheless, our results enabled us to take the mass shift of the modification and the additional site of crosslink formation into consideration when analyzing spectra of crosslinked peptide models *de novo.*

### 3.2 Formation of disulfide, lysinoalanine and lanthionine crosslinks

After defining the concurrent modifications that form when the peptides are heat treated at pH 12, we determined whether crosslinks form between the linear peptides when mixed together under the same treatment. We mixed the three peptides LLSLR, LLKLF and LKDECFR in equimolar pairwise and one triplet combination at either pH 6, 8, 10, or 12, and heated at 70 °C for 1, 2, 4, 6, or 12 days. The mass spectra were then analyzed *de novo* in four steps: 1) identify disulfide crosslinks using a known fragmentation pattern (Section 3.3); 2) identified disulfide ions were then used as a reference to assign lysinoalanine and lanthionine crosslinks to peaks in the spectra of mixtures of peptides (Section 3.4); 3) determine the lysinoalanine crosslinks fragmentation pattern (Section 3.5); and 4) identify trends in crosslink formation as the pH and incubation time increased (Section 3.6).

Identification of all crosslinks was carried out by comparing the observed mass to the theoretical mass of crosslinked peptides, including the mass shift caused by the treatment-induced modification to arginine on the LLSLR peptide observed in the earlier experiment. A discrete database of peptides crosslinked by disulfide, lysinoalanine and lanthionine was prepared. Comparing the theoretical masses of crosslinked peptides to MALDI-TOF MS spectra enabled identification of disulfide and subsequently, lysinoalanine and lanthionine crosslinks in the mixtures of peptides. Sequences of the constituting peptides were confirmed *de novo* sequencing from MS/MS spectra with the identification of at least *x* number of *b* or *y* ions, where *x* is the number of residues in the smaller of the two peptides.

Using synthetic peptides of a known mass and including concurrent modifications observed in earlier experiments minimized the complexity of identifying crosslinks in spectra. The primary challenge when identifying process-induced crosslinks in mass spectra is that typically we do not know which peptides are linked together and what the corresponding mass of crosslinked peptides is. Consequently, every pair of potentially crosslinked peptides needs to be considered when searching a database, leading to a computational explosion. Our approach of using synthetic peptides of a known sequence and mass greatly reduces this challenge, enabling us to easily identify lysinoalanine and undertake a characterization study.

### 3.3 Disulfide crosslinks are confirmed by disulfide triplet cluster

**Supplementary figure 2:**
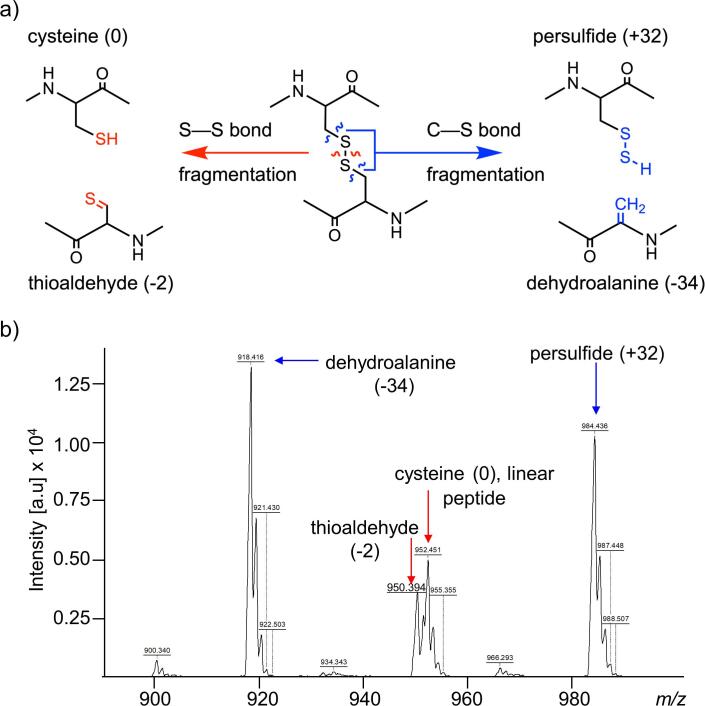


In the first step of analysis, we identified disulfide crosslinks in MALDI-TOF MS spectra of peptide mixtures. Disulfide crosslinks, which can be readily identified by mass spectrometry analysis software, have the advantage of being identifiable by ‘disulfide triplet marker ions’ when fragmented (Goyder et al., 2013). Using this characteristic disulfide triplet cluster, we manually confirmed identification of a disulfide between LKDE**C**FR – LKDE**C**FR (where the **bold** residues are crosslinked and shown as the original reside of the peptide) (*m*/*z* 1901.89) in MS spectrum with identification of the disulfide triplet marker ions in MS/MS spectrum (Fig. S2). These fragmentation marker ions enabled us to confidently identify disulfide crosslinks manually without the use of an analytical software. Using the same workflow to identify lysinoalanine provided an assurance that identification process was valid. Further, the identified disulfide peaks provided an important reference when assigning peaks to the less common, and more challenging, lysinoalanine and lanthionine crosslinks.

### 3.4 Lysinoalanine and lanthionine are distinguished by a modification exclusive to lysinoalanine

Following assignment of disulfide peaks, we then identified lysinoalanine and lanthionine crosslink peaks in the MALDI-TOF MS spectra of the peptide mixtures that had been heated at high pH (Table 1). Lysinoalanine crosslinks were identified at *m*/*z* 1256.8 (between LL**S**LR_(modified)_ – LL**K**LF or LLSL**R**_(modified)_ – LL**K**LF), *m*/*z* 1591.9 (between LKDE**C**FR – LL**K**LF), *m*/*z* 1836.0 (between L**K**DE[DHA]FR – LKDE**C**FR) and potentially at *m*/*z* 1869.9 (between L**K**DECFR – LKDE**C**FR) (Table 1a). Disulfide crosslinks were identified at every pH condition when treated for one or two days, but not at pH 11 and pH 12 at 4 days and above (Table 1b). Lysinoalanine and lanthionione crosslinks were identified at every time interval at pH 11 and pH 12. Lysinoalanine crosslinks were also identified at *m*/*z* 1256.8 and *m*/*z* 1869.9 at pH 10 at every time interval. Crosslinks had to be identified in both duplicate samples to qualify.

**Table 1:** Disulfide, lysinoalanine and lanthionine crosslinks identified in model peptides at different conditions. a) Crosslinks identified when linear peptides LLSLR, LLKLF and LKDECFR are mixed in equimolar pairwise combinations and one triplet combination and heated at 70 °C at either pH 6, 8, 10, or 12, for 1, 2, 4, 6, or 12 days. Sequences of the contributing peptides were confirmed by MS/MS with identification of at least *x* number of *b* or *y* ions, where *x* is the number of residues in the smaller of the two peptides. b) Crosslinks were present when both duplicates were identified after heating the sample at 70 °C at either pH 6, 8, 10, 11 or 12. Samples were analysed at days 1, 2, 4, 6, and 12.

**a)**	**crosslink**	**Theoretical *m*/*z***	**Observed *m*/*z***	**constituting peptides that are crosslinked**^a^
	disulfide	1901.8	1901.9	LKDE**C**FR – LKDE**C**FR
	lysinoalanine	1256.8	1256.8	LL**S**LR_(modified)_ – LL**K**LF b, or LLSL**R**_(modified)_ – LL**K**LF
	lysinoalanine	1591.9	1591.9	LKDE**C**FR – LL**K**LF
	lysinoalanine or lanthionine	1869.9	1869.9	L**K**DECFR – LKDE**C**FR, or LKDE**C**FR – LKDE**C**FR
	lysinoalanine	1835.9	1836.0	L**K**DE[DHA]FR – LKDE**C**FR c

**b)**	**crosslink**	**Days where crosslink is detected**				
						

	disulfide *m*/*z* 1901.8	1,2,4,6 & 12	1,2,4,6 & 12	1,2,4,6 & 12	only 1 & 2	only 1 & 2
	lysinoalanine *m*/*z* 1256.8	–	–	1,2,4,6 & 12	1,2,4,6 & 12	1,2,4,6 & 12
	lysinoalanine *m*/*z* 1591.9	–	–	–	1,2,4,6 & 12	1,2,4,6 & 12
	lysinoalanine or lanthionine *m*/*z* 1869.9	–	–	1,2,4,6 & 12	1,2,4,6 & 12	1,2,4,6 & 12
	lysinoalanine *m*/*z* 1835.9	–	–	–	1,2,4,6 & 12	1,2,4,6 & 12

a Amino acids are referred to by one-letter codes. Cysteine, serine, and lysine residues are original residues of peptide (as discussed in text), constituting crosslinks are in **bold**.

b Modification at arginine as discussed in text.

c [DHA] dehydroalanine as discussed in text.

One of the drawbacks of identifying crosslinks by theoretical mass is that it does not distinguish between different types of crosslinks that have the same mass. For example, the theoretical *m*/*z* of two LKDECFR peptides crosslinked by either lanthionine or lysinoalanine is the same (*m*/*z* 1869.9). This is because the first step in the formation of both crosslinks is the same and this is where the mass loss occurs. In the in the first step, β-elimination occurs at cystine with the loss of H_2_S, forming a carbon–carbon double bond at the DHA intermediate on one peptide (LKDE[DHA]FR) (Fig. 1). In the second step that determines what type of crosslink is formed, the carbon–carbon bond on the DHA of the first peptide reacts with either the SH on cystine of the second peptide to form lanthionine (LKDE**C**FR – LKDE**C**FR) or with the NH_2_ on lysine of the second peptide to form lysinoalanine (LKDE**C**FR – L**K**DECFR). As such, the MS/MS spectra of the *m*/*z* 1869.9 peak that was fragmented contained ions common to both lysinoalanine and lanthionine and we were unable to discriminate between the two crosslinks.

**Supplementary figure 3:**
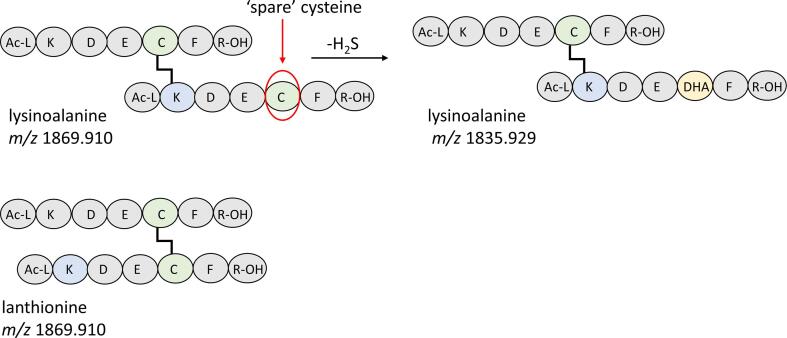


To address this problem, a mass shift that results from the formation of the intermediate DHA was used to identify a lysinoalanine crosslinks at *m*/*z* of 1835.9 in the MS spectrum. Both lysinoalanine and lanthionine crosslinks form *via* the intermediate DHA (Fig. 1). However, only peptides linked by lysinoalanine have a ‘spare’ cysteine available that can also form DHA (L**K**DE[DHA]FR – LKDE**C**FR, (Fig. S3,  arrow). In this case, the cysteine residues of one peptide forms DHA and goes on to react with lysine to form lysinoalanine, while the cysteine in the second peptide moiety also forms DHA but does not form a crosslink. Formation of DHA that does not form a crosslink results in the loss of H_2_S on a thiol of cysteine, which leads to a mass shift of Δ33.98 Da. In contrast, in peptides linked by lanthionine (LKDE**C**FR – LKDE**C**FR), the cysteine residues of one peptide moiety forms DHA and goes on to react with cysteine in the second peptide moiety. Once the lanthionine crosslink is formed, there are no free thiol groups and DHA does not form again. Consequently, when lysinoalanine forms, a modification and a mass shift can occur on the peptide that cannot occur when lanthionine forms.

In summary, by subjecting three synthetic linear peptides to stressors of high pH at 70 °C for varying lengths of time, disulfide, lysinoalanine and lanthionine crosslinks were formed, identified in MALDI-TOF MS spectra and confirmed in MS/MS spectra (Table 1). The simple nature of the models enables lanthionine and lysinoalanine crosslinks, which cannot be distinguished in MS/MS spectra, to be differentiated by a convenient Δ33.98 Da mass shift exclusive to the lysinoalanine model. While these models are simple, discriminating between lysinoalanine and lanthionine is not elementary and highlights the difficulties in identifying these crosslinks in complex food samples.

### 3.5 Lysinoalanine is characterized by a fragmentation pattern

**Fig. 3:**
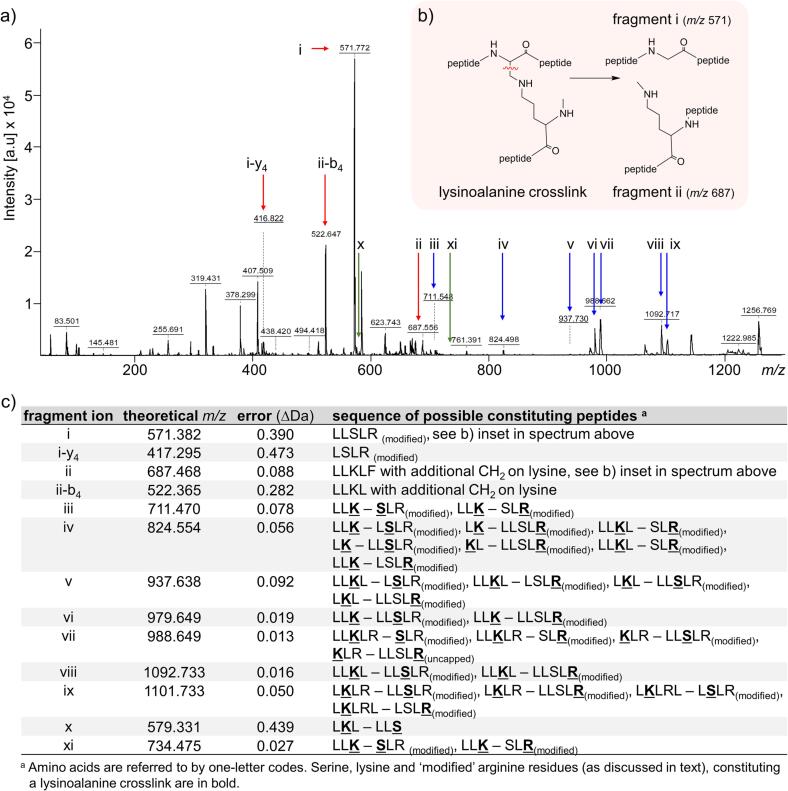
MS/MS spectra of lysinoalanine crosslink between LLSLR and LLKLF. a) MALDI MS/MS spectrum over a range of *m*/*z* 50–1250 of a lysinoalanine crosslink between the LLSLR_(modified)_ and LLKLF peptides (*m*/*z* 1256.82) formed after heating at 70 °C at pH 12 for 2 days. Fragment ion i, shown with a  arrow (*m*/*z* 571.772), is the breakdown product of the LLSLR_(modified)_ moiety when the lysinoalanine crosslink breaks at α-β carbon and fragment ion ii shown with a  arrow (*m*/*z* 687.556), the corresponding breakdown product of the LLKLF moiety. Fragment ion i-y_4_ (*m*/*z* 416.822) is the y_4_ ion of fragment i, and fragment ion ii-b_4_ (*m*/*z* 522.647) is the b_4_ ion of fragment ion ii. Fragment ions iii–ix shown with  arrows result from peptides crosslinked by lysinoalanine, where the position of the crosslink on the LLSLR_(modified)_ peptide could not be confirmed as being at the serine or the arginine residues. The fragment ions x and xi shown with  arrows results from peptides crosslinked by lysinoalanine between lysine in one peptide and serine (*via* dehydroalanine) in the other peptide. b) (inset) Fragmentation mechanism of lysinoalanine crosslink between LLSLR_(modified)_ and LLKLF at the α-β carbon that forms fragmentation ion i and fragmentation ion ii. c) Fragment ions and sequence of possible constituting peptides. (For interpretation of the references to colour in this figure legend, the reader is referred to the web version of this article.)

**Fig. 4:**
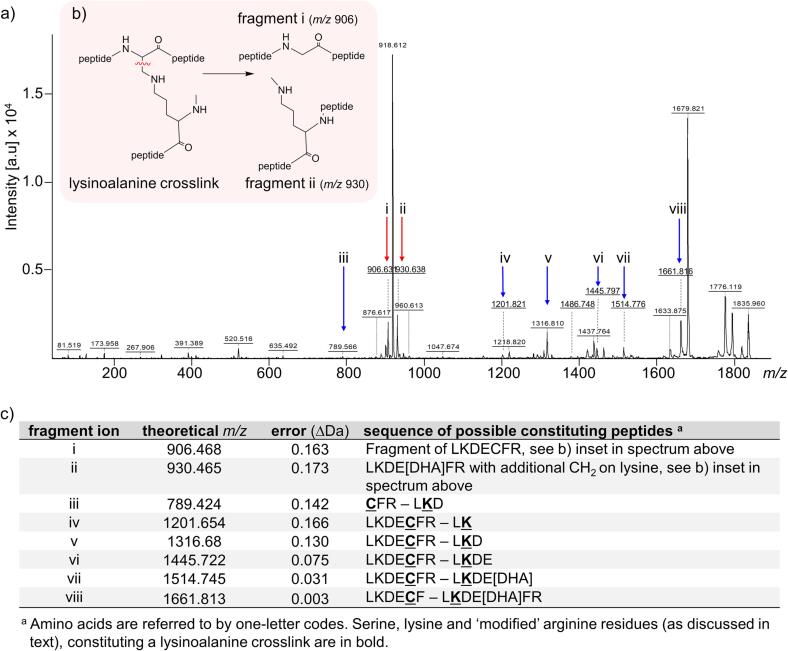
MS/MS spectra of lysinoalanine crosslink between two peptides of LKDECFR. a) MALDI MS/MS spectrum over a range of *m*/*z* 100–1900 of the lysinoalanine crosslink between two peptides of LKDECFR with *m*/*z* 1835.924. The crosslink was formed after heating at 70 °C at pH 12 for 6 days. Fragment ion i, shown with a  arrow (*m*/*z* 906.631), is the breakdown product of the LKDECFR moiety when lysinoalanine crosslink breaks at α-β carbon. Fragment ion ii, shown with a  arrow (*m*/*z* 930.465), is the corresponding breakdown product of the LKDE[DHA]FR moiety. Fragment ions iii–viii, shown with a  arrow, constitute fragments of crosslinked peptides where the lysinoalanine crosslink remains intact. b) (inset) Fragmentation mechanism of lysinoalanine crosslink between LKDECFR and LKDE[DHA]FR at the α-β carbon that forms fragmentation i and fragmentation ii. c) Fragment ions and sequence of possible constituting peptides. (For interpretation of the references to colour in this figure legend, the reader is referred to the web version of this article.)

Using the lysinoalanine crosslinks identified in MALDI-TOF MS spectra we were able to characterize the MS/MS spectra and look for a pattern in the fragmentation that might expose a diagnostic ion for lysinoalanine. Unlike disulfide crosslinks that can be detected in an automated way and have a characteristic triplet cluster, a characteristic ion or fragmentation pattern for lysinoalanine has yet to be identified and incorporated into search algorithms. Fragmentation using electrospray ionisation (ESI) higher-energy collision dissociation mass spectrometry favors cleavage between the ε-carbon and the adjacent nitrogen atom in the lysinoalanine crosslink (Rombouts et al., 2016). From our analysis of MALDI MS/MS spectra, we identified a characteristic fragmentation between the α-carbon and β-carbon in lysinoalanine crosslinks between LL**S**FR_(modified)_ – LL**K**LF and LKDE**C**FR – LL**K**LF (Fig. 3 and Fig. 4) formed over a range of stress conditions, as detailed below. Differences in crosslink fragmentation patterns with ESI and MALDI mass spectrometers are unsurprising given the ways in which energy is added to the ions to induce fragmentation are completely different (Aebersold & Mann, 2003).

i. Fragmentation of lysinoalanine between LLSLR and LLKLF

As observed in earlier experiments, the concurrent modification on the terminal arginine introduces a second potential site where a lysinoalanine crosslink can potentially form on the LLSLR peptide; either between serine (*via* dehydroalanine) and lysine or between the ‘modified’ arginine and lysine. The location of the crosslink was determined before characterizing the lysinoalanine crosslink between LLSLR and LLKLF at *m*/*z* 1256.82. The position of the lysinoalanine crosslink was observed between serine (*via* dehydroalanine) and lysine (LL**S**LR_(modified)_ – LL**K**LF) from the MALDI MS/MS spectrum (*m*/*z* 1256.82) (Fig. 3a). Fragment ions x and xi in Fig. 3a (highlighted with  arrows) constitute a lysinoalanine crosslink between serine (*via* dehydroalanine) and lysine and are the key fragments to distinguish the location of the crosslink. While ions x and xi are of low abundance, no fragments for a crosslink between the modified arginine and lysine (LLSL**R**_(modified)_ – LL**K**LF) are found.

Once the location of the lysinoalanine crosslink was confirmed, further ions in the MALDI MS/MS spectrum of *m*/*z* 1256.82 were assigned to fragments of the crosslinks. Breaking bonds in the lysinoalanine crosslink, as opposed to breaking peptide bonds between residues in the linear peptide moieties of the crosslink (typical *b* and *y* ion breakages), provides a means to characterize the crosslinks.

**Supplementary figure 4:**
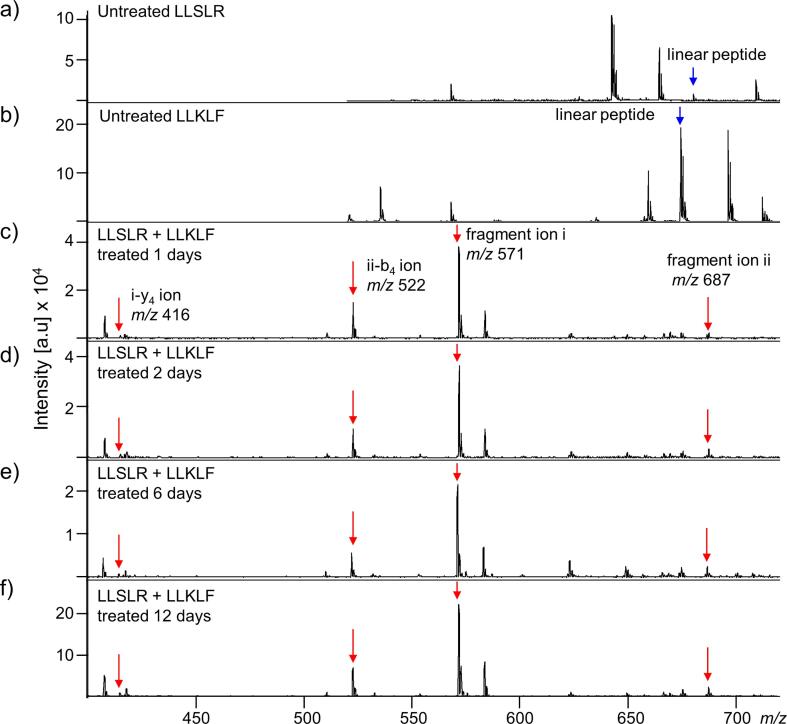


We observed fragmentation between the α-carbon and β-carbon of a lysinoalanine crosslink between LL**S**LR_(modified)_ – LL**K**LF in the MALDI MS/MS spectrum (*m*/*z* 1256.82) (Fig. 3a). Cleavage at this carbon–carbon bond in the lysinoalanine crosslink produces the breakdown products in Fig. 3b that are identified as fragment i and ii, highlighted with a  arrow in MALDI MS/MS spectrum in Fig. 3a. Fragment ion i, shown with a  arrow at *m*/*z* 571.772 (Δ0.390 Da), being the most intense peak, is the breakdown product of the LLSLR_(modified)_ moiety of the crosslink. Fragment ii, shown with a  arrow at *m*/*z* 687.556 (Δ0.088 Da), is the corresponding breakdown product of the LLKLF moiety of the crosslink which has an additional CH_2_ group on the ε-amino group of lysine. Fragment ion i-y_4_ (*m*/*z* 416.822, Δ0.473) is the y_4_ ion of fragment i, and ii-b_4_ (*m*/*z* 522.647, Δ0.282 Da) is the b_4_ ion of fragment ion ii. Fragment ions i, ii and i-y_4_, and ii-b_4_ are not detected in the MS spectra of the two linear peptides moieties of the crosslink model (Fig. S4). Fragment ions iii–ix, shown with  arrows in Fig. 4a, result from peptide bond breakages along the linear peptide portions of the crosslink when the crosslink remains intact. In these fragments, the location of the crosslink, either between serine (*via* dehydroalanine) and lysine or between the ‘modified’ arginine and lysine, could not be confirmed. Fragment ions x and xi, highlighted with  arrows, confirm the lysinoalanine crosslink is between serine (*via* dehydroalanine) and lysine. The observed *m*/*z* values of each of the fragments correspond with varying error to the theoretical *m*/*z* (Fig. 4c).

ii. Fragmentation of lysinoalanine between LKDECFR and LKDECFR

**Supplementary figure 5:**
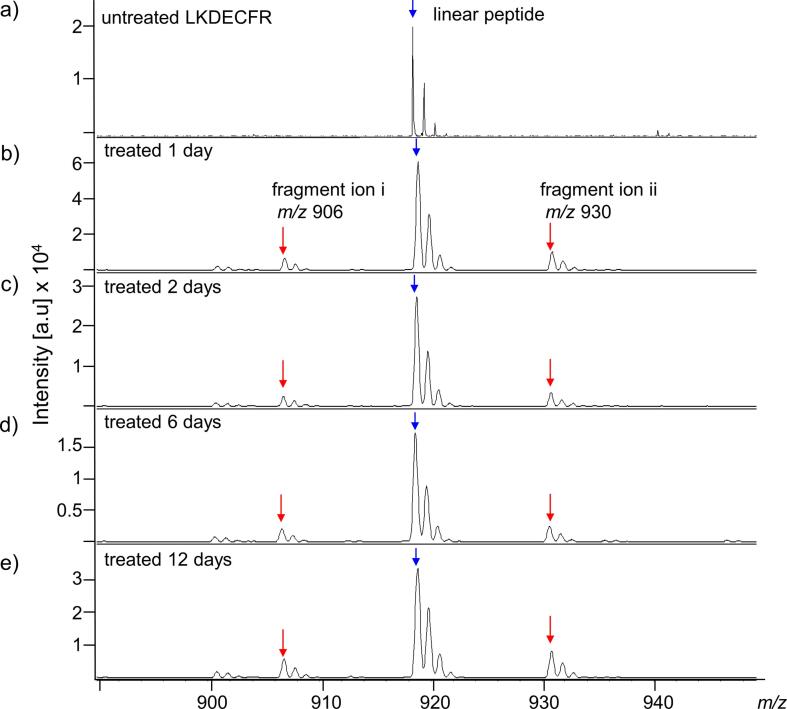


Fragmentation between the α-carbon and β-carbon of a lysinoalanine crosslink was also observed in the MALDI MS/MS spectrum of a lysinoalanine crosslink between LKDECFR and LKDECFR (Fig. 4a). In this model the ‘spare’ cysteine residue not involved in the lysinoalanine crosslink has undergone β-elimination to form dehydroalanine (LKDE[DHA]FR) (*m*/*z* 1835.924). Fragment ion i, shown with a  arrow at *m*/*z* 906.631 (Δ0.163), is one of the breakdown products when the lysinoalanine crosslink breaks at α-β carbon (Fig. 4b). Fragment ion ii, shown with a  arrow (*m*/*z* 930.465 (Δ0.173), is the corresponding breakdown product, having an additional CH_2_ group on the ε-amino group of lysine of the LKDE[DHA]FR (Fig. 4b). Fragment ions i and ii are not detected in the MS spectra of the two linear peptides moieties of the crosslink (Fig. S5). Fragment ions iii–viii, shown with a  arrow in Fig. 4a, constitute fragments of crosslinked peptides where the lysinoalanine crosslink remains intact. The observed *m*/*z* values of each of the fragments correspond within 0.18 Da of the theoretical *m*/*z* (Fig. 4c).

In summary, despite a concurrent modification on the terminal arginine, which introduces an additional site of lysinoalanine crosslinking, we were able to establish the location of a lysinoalanine crosslink between LLSLR and LLKLF using fragmentation ions in the MALDI MS/MS. We then located a putative fragmentation pattern resulting from cleavage between the α-carbon and β-carbon of a lysinoalanine crosslink in two crosslinks models.

The synthetic peptide models used here provide an initial simplified view into the characterization of lysinoalanine which are extremely difficult to analyze in food samples. Many of the challenges present during general bottom-up proteomics workflows on real food samples are avoided by the models. Aggregation caused by crosslinking which reduces solubility and hinders protein extraction was bypassed (Lebendiker & Danieli, 2014) and digestion enzymes where not required. Avoiding digestions enzymes is attractive, because in the case of trypsin, it is ineffective when the cleavage site (either the carboxyterminal of arginine or lysine resides), coincides with the residue involved in crosslinking, as is the case with lysinoalanine (Friedman, 1999, Possompes and Berger, 1986). The models also help to avoid the need for enriching crosslinked peptides, which are in low abundance compared to linear peptides.

### 3.6 More lysinoalanine forms with increasing time and pH

Given heat and alkaline conditions favor lysinoalanine formation (Friedman, 1999, Zhao et al., 2016), we next investigated the influence of pH and length of heating on crosslink formation in the model crosslinks. In one series of treatments that varied pH, the LKDECFR peptide was heated at 70 °C at either pH 6, 8, 10, 11, or 12 for 1 day. In a second series of treatments that varied heating time, the LKDECFR peptide was heated at 70 °C at pH 12 from 3 h to 12 days. We observed a broad increase in the relative intensity of lysinoalanine and a decrease in disulfide crosslinks with increasing pH. The same more defined trend was observed when the length of heating time was increased.

The peak intensity of disulfide and lysinoalanine crosslinks were normalized using the total ion current. Normalizing peak intensity negates the effect of acquisition parameters and focused solely on the change in pH, or incubation time. Because there is no internal standard there is a run-by-run variation between the quantities. While peak intensity is not a quantitative measure, comparing the normalized relative intensities of crosslink peaks provides an overview of trends in crosslink formation.

To assess trends in crosslink formation, we looked at pure sample of the peptide LKDECFR. Although all mixtures of the three peptides were investigated, a pure sample of LKDECFR is a good exemplar as it forms disulfide (*m*/*z* 1901.89) and lysinoalanine (*m*/*z* 1835.9) crosslinks. Furthermore, when examining crosslinks between the same peptide (LKDECFR), the two main factors affecting crosslinking, steric hindrance and structural proximity, are consistent and do not play a role in variances to the system. Consequently, there is a degree of competition between the type of crosslinks that can form depending on the conditions, making samples of LKDECFR a good candidate to analyze.

i. Trends when increasing pH

**Fig. 5:**
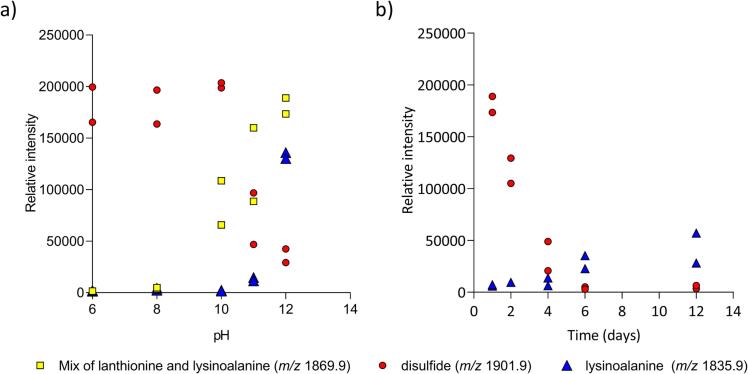
Comparison of lysinoalanine and disulfide formation with changes in pH and time. Relative intensities from replicate experiments of lysinoalanine and lanthionine (*m*/*z* 1869.9,  square), disulfide (*m*/*z* 1901.89,  circle), and lysinoalanine (*m*/*z* 1835.9,  triangle). a) Crosslinks at either pH 6, 8, 10, 11, or 12. b) Crosslinks formed between LKDECFR when heated at 70 °C for 1 day and tracked over time at pH 12. (For interpretation of the references to colour in this figure legend, the reader is referred to the web version of this article.)

**Supplementary figure 6:**
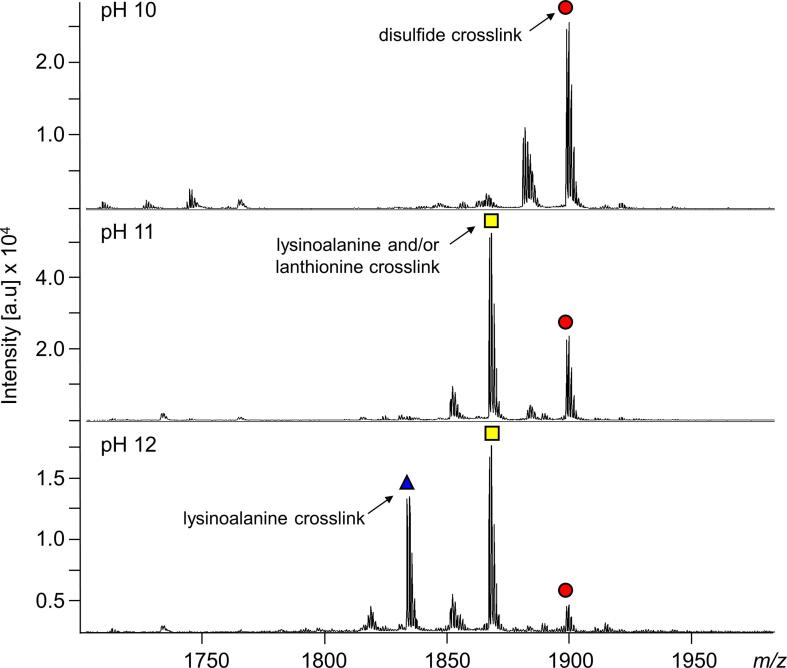


A general increase in the real intensity of lysinoalanine crosslinks and a decrease in disulfide crosslinks is observed with increasing pH when comparing the relative intensity of lysinoalanine, marked with a  triangle (*m*/*z* 1835.9) in Fig. 5a), to disulfide crosslinks, marked with a  circle (*m*/*z* 1901.89). The real intensity of the peaks that are mixture of lysinoalanine and lanthionine, marked with a  square (*m*/*z* 1869.9) (as discussed in section 3.4 above), also increases above pH 10. The trend in changing peak intensity is most apparent at pH 10, 11 and 12, and can be observed when directly comparing the spectra at these pH values (Fig. S6). This observation suggests the higher the pH, the more lysinoalanine will be present compared to disulfide crosslinks. The correlation of lysinoalanine formation with pH is well established with soybean studies, showing lysinoalanine appearing at pH 9 and increasing continually to pH 12.5 before decreasing at pH 13.9 (Friedman et al., 1984). This is unsurprising, since the pK_a_ value of the ε-amino group of lysine is near 10 in most proteins and complete ionization does not occur until pH 12 (Friedman, 1999, Friedman et al., 1982). However, a direct correlation between increasing lysinoalanine and decreasing disulfide crosslinks with pH, as implied here, has not been previously defined. Greater cleavage of disulfide crosslinks at pH 11 compared to pH 9, resulting in more free thiol groups that can then be converted to dehydroalanine, has been observed (Monahan et al., 1995, Nashef et al., 1977). Increasing concentrations of dehydroalanine will likely lead to the relative increase in the formation of lysinoalanine in the absence of steric hindrance factors and with high concentrations of reactive ionized NH_2_ groups present at pH 12. Consequently, newly formed dehydroalanine, which reacts with the ε-amino of lysine to form lysinoalanine, prevents reversion back to the disulfide. From these results, we suggest an increase in the cleavage of disulfide crosslinks is a likely contributor to the increased formation of lysinoalanine.

Many foods will be affected by the relationship between lysinoalanine formation and disulfide bond cleavage as a result of alkaline treatment, including Chinese noodles, pretzels and crackers, which use high pH during preparation (Hasegawa et al., 1987, Rombouts et al., 2012). A greater understanding of lysinoalanine and crosslink formation is particularly important in the development of protein alternatives made from whey and soy protein isolates. Protein isolates can be extruded from whey using high alkaline conditions, resulting in increased denaturation and stringy texture that is attractive to meat applications (Onwulata et al., 2006).

ii. Trends when increasing length of heating

**Supplementary figure 7:**
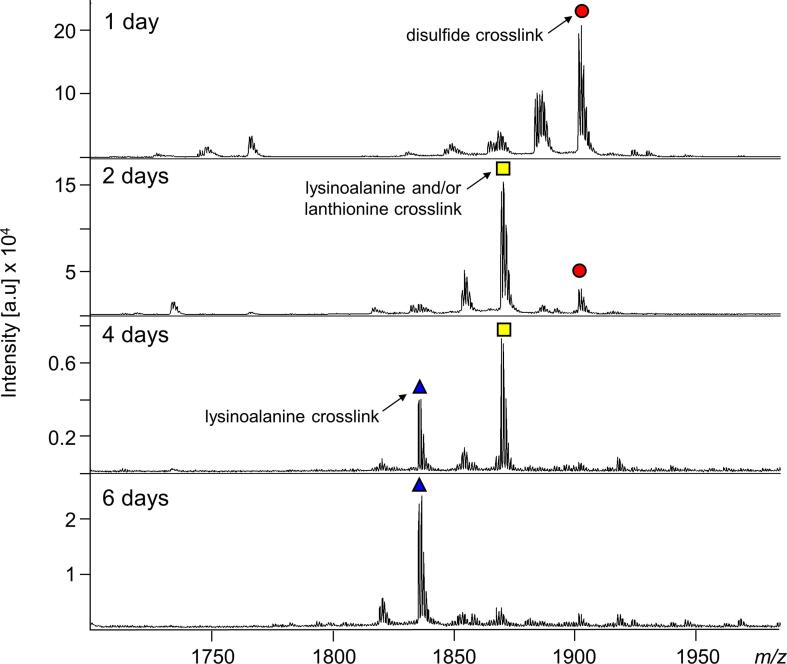


An increase in lysinoalanine crosslinks and a decrease in disulfide crosslinks was also observed when LKDECFR was heated at 70 °C at pH 12 for increasing lengths of time. The relative intensity of lysinoalanine ( triangle (*m*/*z* 1835.9) in Fig. 5b) increases consistently to day 12, while disulfide crosslinks (red circle (*m*/*z* 1901.89) in Fig. 5b) drops away. This trend of increased lysinoalanine formation from degraded disulfide with longer heating time was also seen when comparing the real un-normalized intensities of the crosslinks (Fig. S7). While we had expected longer exposure to heating to favor lysinoalanine formation (Friedman et al., 1984), we did not expect it to continue to increase for such an extended period. Increasing formation of lysinoalanine crosslinks in soybeans heated in 0.1 N NaOH at 75 °C, measured by amino acid analysis, has been observed for up to three hours before formation plateaued (Friedman et al., 1984). We were surprised to observe continued formation of lysinoalanine in the peptide model over the twelve days of heating at pH 12 at 70 °C without levelling off. This may be a due to differences between peptide samples as opposed to protein samples. In the case of peptides, residues have greater solvent exposure and are not ‘protected’ in the same way as some regions of a protein buried within the tertiary structure of a protein. Anomalies between the different techniques used to measure crosslink quantities (amino acid analysis compared to comparing normalized peak intensities) and different heating temperatures (75 °C and 70 °C) prevents direct comparison of the contrasting observations. Further, the influence of heating time may be different with the type and source of the sample. However, our observations generally suggest that heating time may have greater influence when longer periods are considered in peptides. This is particularly important in food processing, considering the vast variety of food proteins and that significant amounts of lysinoalanine appeared in soybean under relatively mild pH, time and temperature conditions (Friedman et al., 1984).

The increase in the relative intensity of lysinoalanine crosslinks with longer incubation time appears to occur at the expense of disulfide bonds, Fig. 5b. This observation suggests that, as with increased pH, increased heating time contributes to the breaking of disulfide bonds. Cleaved disulfide bonds releases thiol groups that form dehydroalanine which react with lysine to form lysinoalanine.

In summary, the trends in relative intensity of crosslink peaks suggest that the longer a peptide is heated and the greater the pH (up to pH 12), the more lysinoalanine forms from cleaved disulfide crosslinks. This has implications for a range of foods, particularly protein alternatives that use protein isolates sourced from soy and whey and alkaline conditions.

## 4 Conclusion

The aim of the research is to characterize the spectra of lysinoalanine crosslinks formed over range of time and pH conditions. We present here novel signature ions for lysinoalanine following fragmentation between the α-carbon and β-carbon of the crosslink in MALDI MS/MS spectra. These ions provide a diagnostic that can reduce the complexity of analyzing spectra and is the first step in automated mapping of the lysinoalanine crosslink. We observed increases in lysinoalanine formation at pH 12 to 12 days, that is longer than previously reported. Lysinoalanine formation increased with a corresponding reduction in disulfide crosslinks. A greater knowledge of crosslink formation is crucial to the development of protein alternatives that use alkaline treatment to extract protein. Characterizing lysinoalanine is essential for understanding the health implications in many foods and increasing nutritional value.

## Funding

This work was supported by generous support of AgResearch and the University of Canterbury for the Connect Scholarship, and funding from the Riddet Institute.

## CRediT authorship contribution statement

**Hannah McKerchar:** Methodology, Investigation, Formal analysis, Writing – original draft, Writing – review & editing, Visualization. **Jolon M. Dyer:** Writing – review & editing. **Juliet A. Gerrard:** Writing – review & editing. **Evelyne Maes:** Methodology, Investigation, Writing – review & editing. **Stefan Clerens:** Resources, Writing – review & editing. **Renwick C.J. Dobson:** Supervision, Funding acquisition, Writing – review & editing.

## Declaration of Competing Interest

The authors declare that they have no known competing financial interests or personal relationships that could have appeared to influence the work reported in this paper.

## Data Availability

Data will be made available on request.
