# Supplementary material for: Characterizing lysinoalanine crosslinks in food systems: Discovery of a diagnostic ion in model peptides using MALDI mass spectrometry
Source: Food Chem X. 2023 Jul 23;19:100800. doi: 10.1016/j.fochx.2023.100800 (PMC10534164; doi:10.1016/j.fochx.2023.100800)
Supplement: Supplementary data 1 [file mmc1.docx]

Supporting information for

**Characterizing lysinoalanine crosslinks in food systems: discovery of a diagnostic ion in model peptides using MALDI mass spectrometry**

Hannah MCKERCHAR^a,b,c^, Jolon M. DYER^a,f,g^, Juliet A. GERRARD^a,b,e^, Evelyne MAES^a,b,c^, Stefan CLERENS^a,b,c^, Renwick C. J. DOBSON^a,b,d^

^a^ Biomolecular Interaction Centre, School of Biological Sciences, University of Canterbury, Christchurch 8140, New Zealand

^b^ Riddet Institute, based Massey University, Palmerston North 4442, New Zealand

^c^ Proteins and Metabolites Team, AgResearch Lincoln Research Centre, Lincoln 7608, New Zealand

^d^ Department of Biochemistry and Molecular Biology, Bio21 Molecular Science and Biotechnology Institute, University of Melbourne, Victoria 3010, Australia

^e^ School of Biological Sciences and School of Chemical Sciences, University of Auckland, Auckland, New Zealand

^f^ The New Zealand Institute for Plant and Food Research, Lincoln Research Centre, Lincoln, 7608, New Zealand

^g^ Department of Wine, Food and Molecular Biosciences, Lincoln University, Lincoln, 7647, New Zealand

**Corresponding author:** Prof. Renwick Dobson: [renwick.dobson@canterbury.ac.nz](mailto:renwick.dobson@canterbury.ac.nz); Current address: School of Biological Sciences, University of Canterbury, Private Bag 4800, Christchurch 8140, New Zealand

**
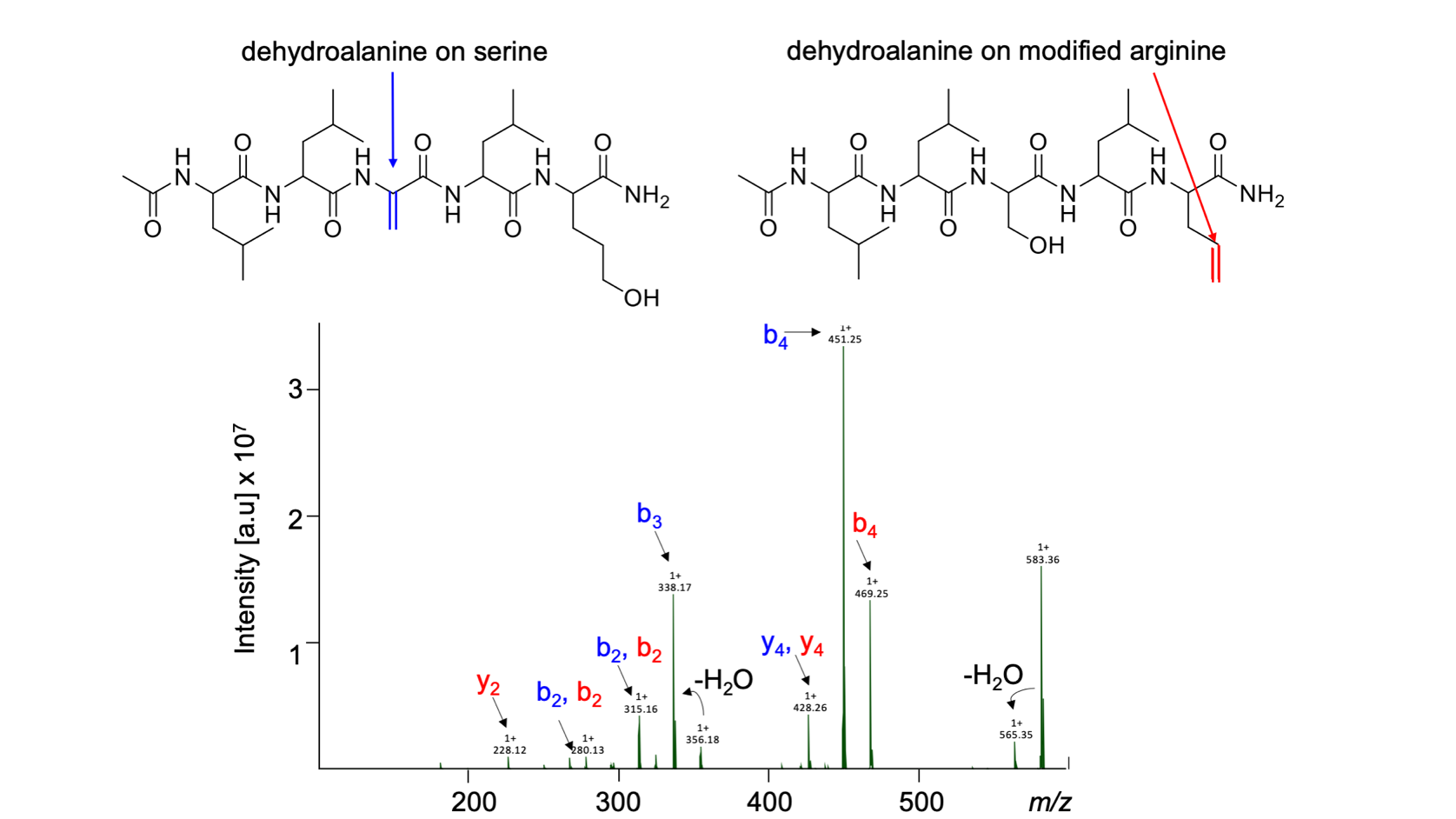
Figure S1. MS/MS spectra of LLSLR_(modified)_.** Off-line nano-electrospray ionisation ion trap MS/MS spectra of LLSLR_(modified)_ *m/z* 583.39, suspended in 0.01 M NaOH at pH 12 and heated for 3 hr at 70 °C, over *m/z* 100–600. Fragments from two species are identified and annotated in the spectra: one where dehydroalanine modification occurs at serine residue (shown in blue); and the other where dehydroalanine modification occurs on the modified arginine residue (shown in red).

**
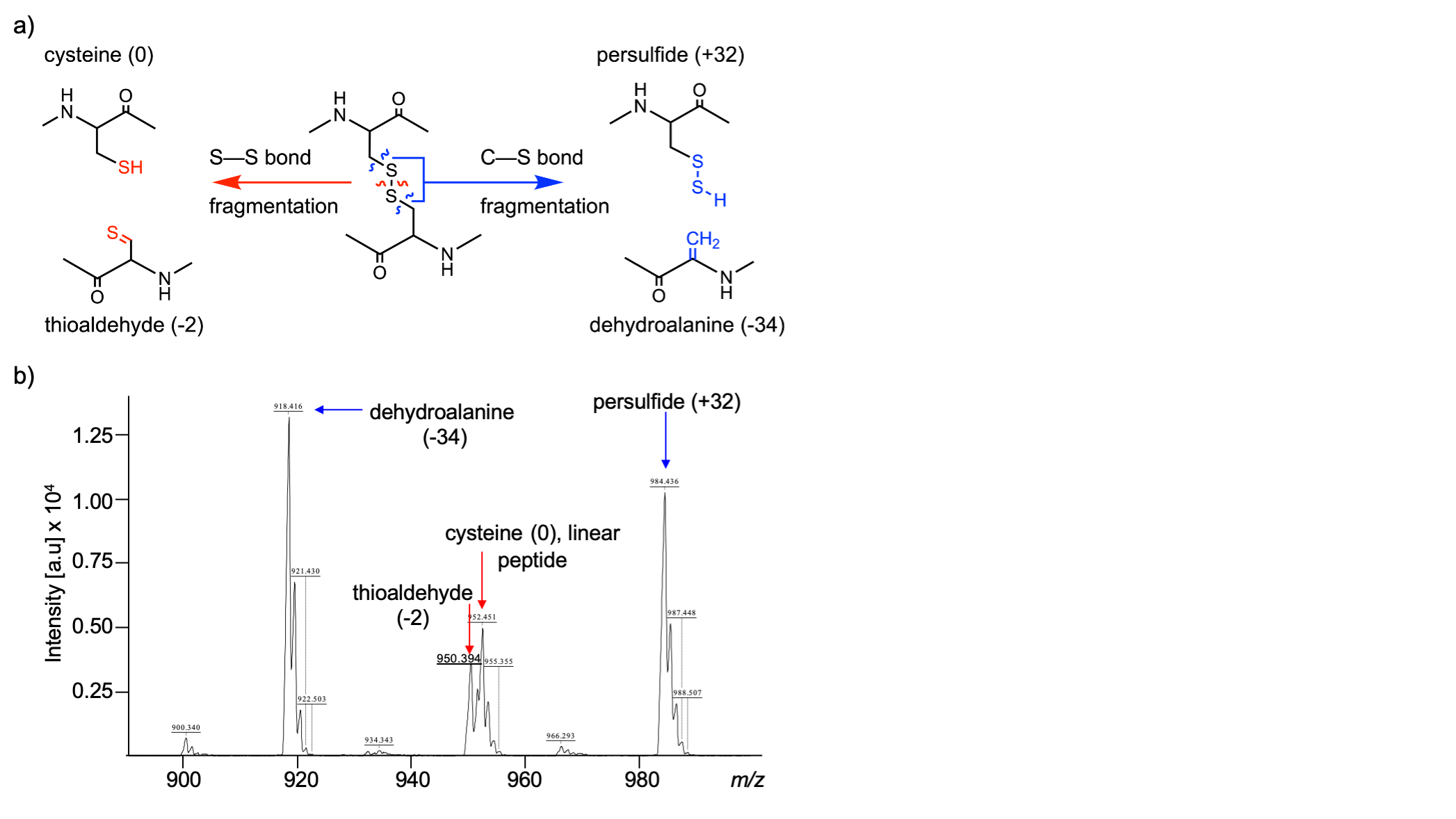
Figure S2. Disulfide crosslinks triplet cluster. a)** Mechanism of fragmentation of disulfide crosslink at sulfur-carbon bond to form linear peptide with a cysteine residue and thioaldehyde or fragmentation at carbon-sulfur bond to form dehydroalanine and persulfide. **b)** MALDI MS/MS spectrum of disulfide crosslink between two LKDECFR peptides with *m/z* 1901.89, over a *m/z* range of 890–1000, from a crosslink formed after heating at 70 °C at pH 12 for 1 day. Linear peptide with cysteine residue at *m/z* 952, thioaldehyde 2 Da less than the linear peptide at *m/z* 950, dehydroalanine 34 Da less at *m/z* 918 and persulfide at 32 Da more at *m/z* 984.

**
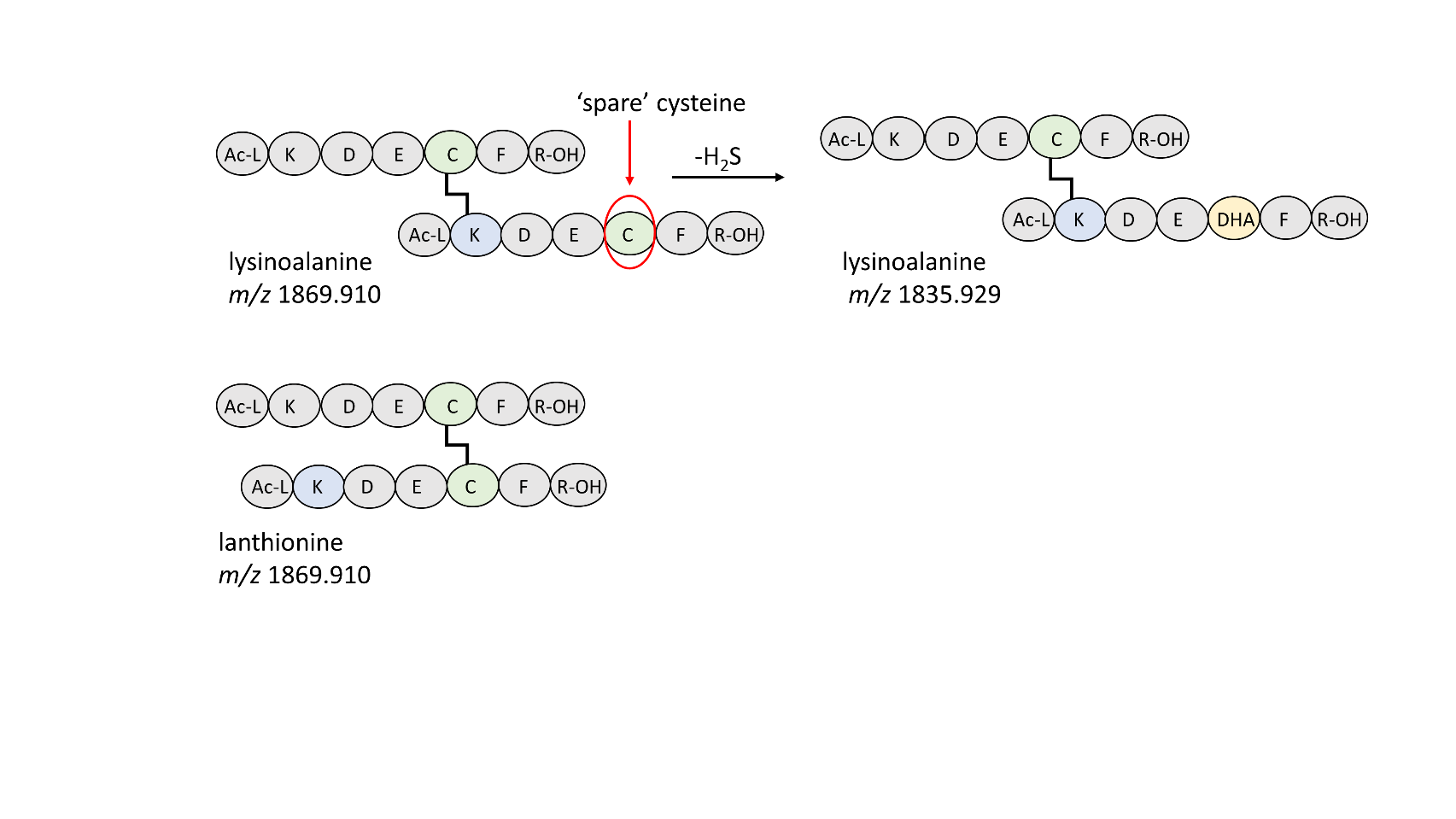
**

**Figure S3. Formation of DHA intermediate used to identify lysinoalanine crosslinks at *m/z* of 1835.9.** Lysinoalanine crosslinks (between C and K) and lanthionine crosslinks (between two Cs) are identified at *m/z* 1869.10. Lysinoalanine crosslinks at *m/z* 1869.10 have a ‘spare’ cystine (red arrow) that can undergo β-elimination (mass shift of 33.98 Da) to form DHA resulting in a lysinoalanine crosslinks at *m/z* 1835.9.

**
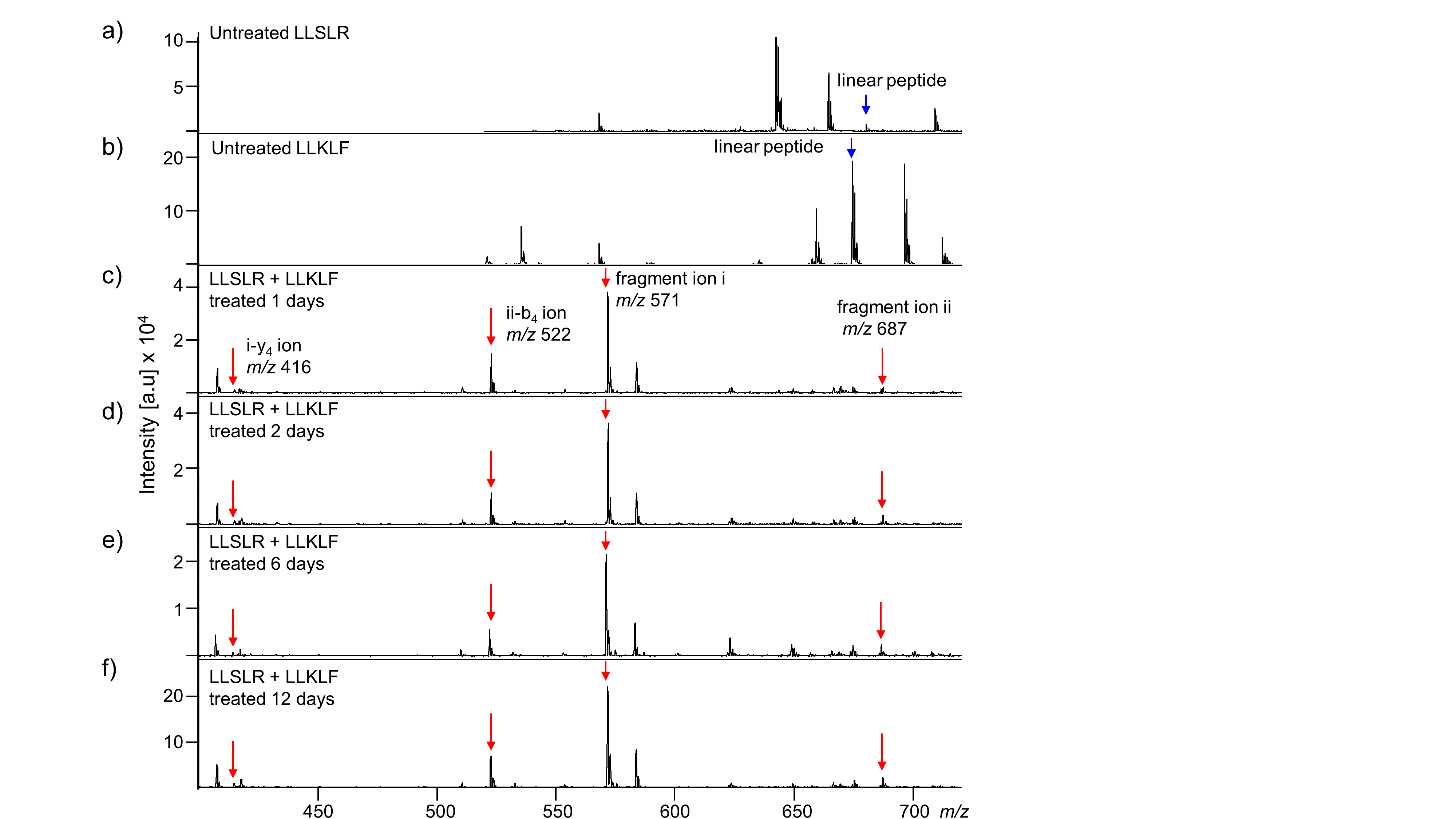
**

**Figure S4. MS spectra of untreated LLSLR and LLKLF without crosslink and MS/MS lysinoalanine crosslink between** **LLSLR and LLKLF**. **a)** MALDI MS spectrum of untreated LLSLR, linear peptide at *m/z* 687 and **b)** MALDI MS spectrum of untreated LLKLF, linear peptide at *m/z* 674. Neither spectra in a) and b) have a lysinoalanine crosslink at *m/z* 1256.82 or ions at *m/z* 687, *m/z* 571, *m/z* 522 and *m/z* 416. **c–f)** MALDI MS/MS spectra of lysinoalanine crosslink between LLSLR_(modified)_ and LLKLF *m/z* 1256.82 treated at pH 12 at 70 °C for one day, two day, six days and 12 days over a range of *m/z* 400–720. The two fragment ions when lysinoalanine crosslink breaks at α-β carbon at *m/z* 571 or *m/z* 687 and the respective y_4_ ion at *m/z* 416 and b_4_ ions at *m/z* 522 appear consistently across all treated samples.

**
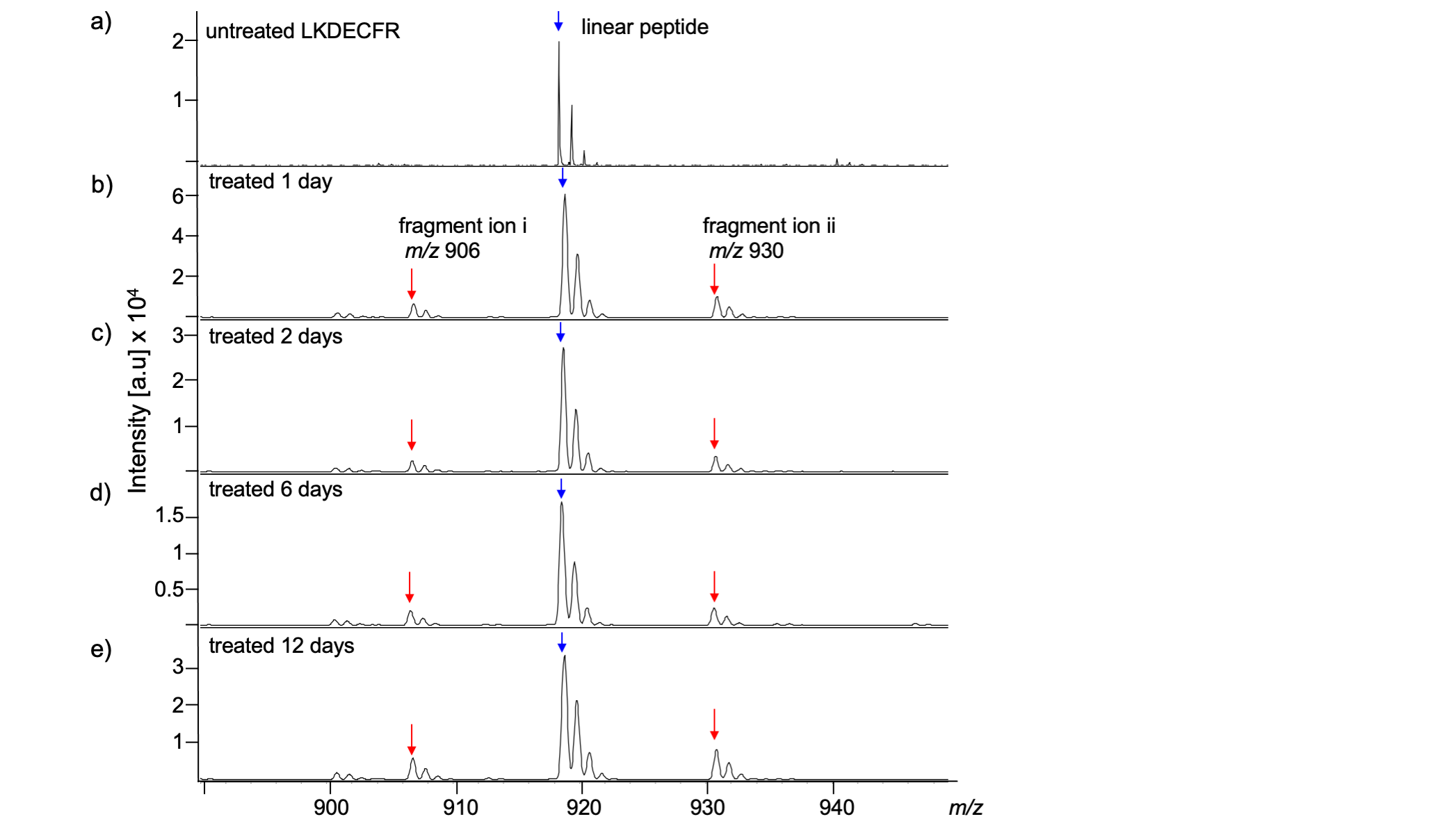
Figure S5. MS spectra of untreated LKDECFR without crosslinks and MS/MS lysinoalanine crosslink between** **two peptides of LKDECFR. a)** MALDI MS spectrum of untreated LKDECFR without crosslink showing the linear peptide at *m/z* 918.73 and no ions at *m/z* 930 or *m/z* 906. **b–e)** four MALDI MS/MS spectra of lysinoalanine crosslink between two peptides of LKDECFR, *m/z* 1835.92, treated at pH 12 at 70 °C for one day (**b**), two day (**c**), six days (**d**), and 12 days (**e**), over an range of *m/z* 890–950. The two fragment ions formed when the lysinoalanine crosslink breaks between the α-β carbons at *m/z* 930 and *m/z* 906 appear consistently across all treated samples.

**
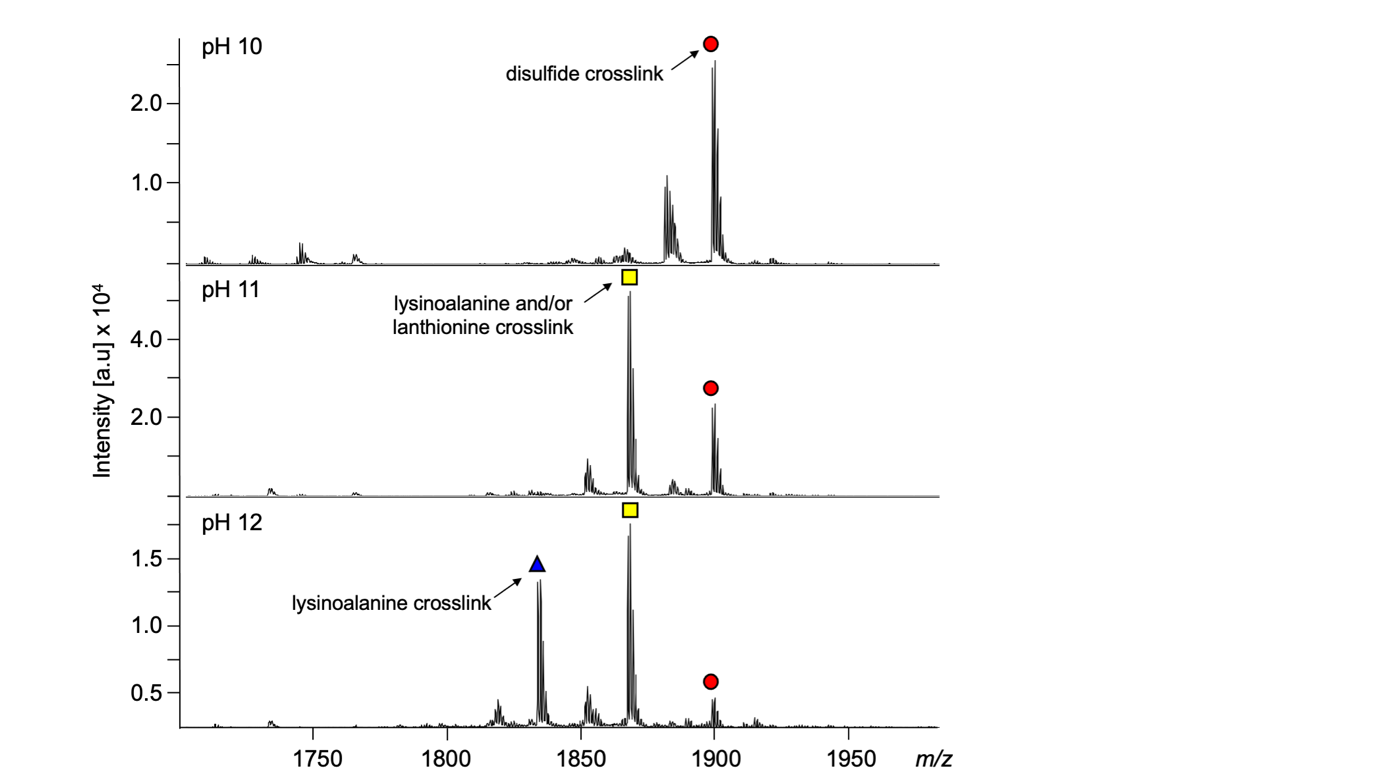
Figure S6. Trend in lysinoalanine and disulfide formation with increasing pH.** MALDI MS spectra of LKDECFR heated at 70 °C for 1 day at either pH 10, 11, or 12, over a *m/z* range of 1700–2000. The disulfide at *m/z* 1901.89 (red circle), a mix of lysinoalanine and/or lanthionine at *m/z* 1869.9 (yellow square), and the lysinoalanine at *m/z* 1835.9 (blue triangle) are observed at varying intensities in each spectrum.**
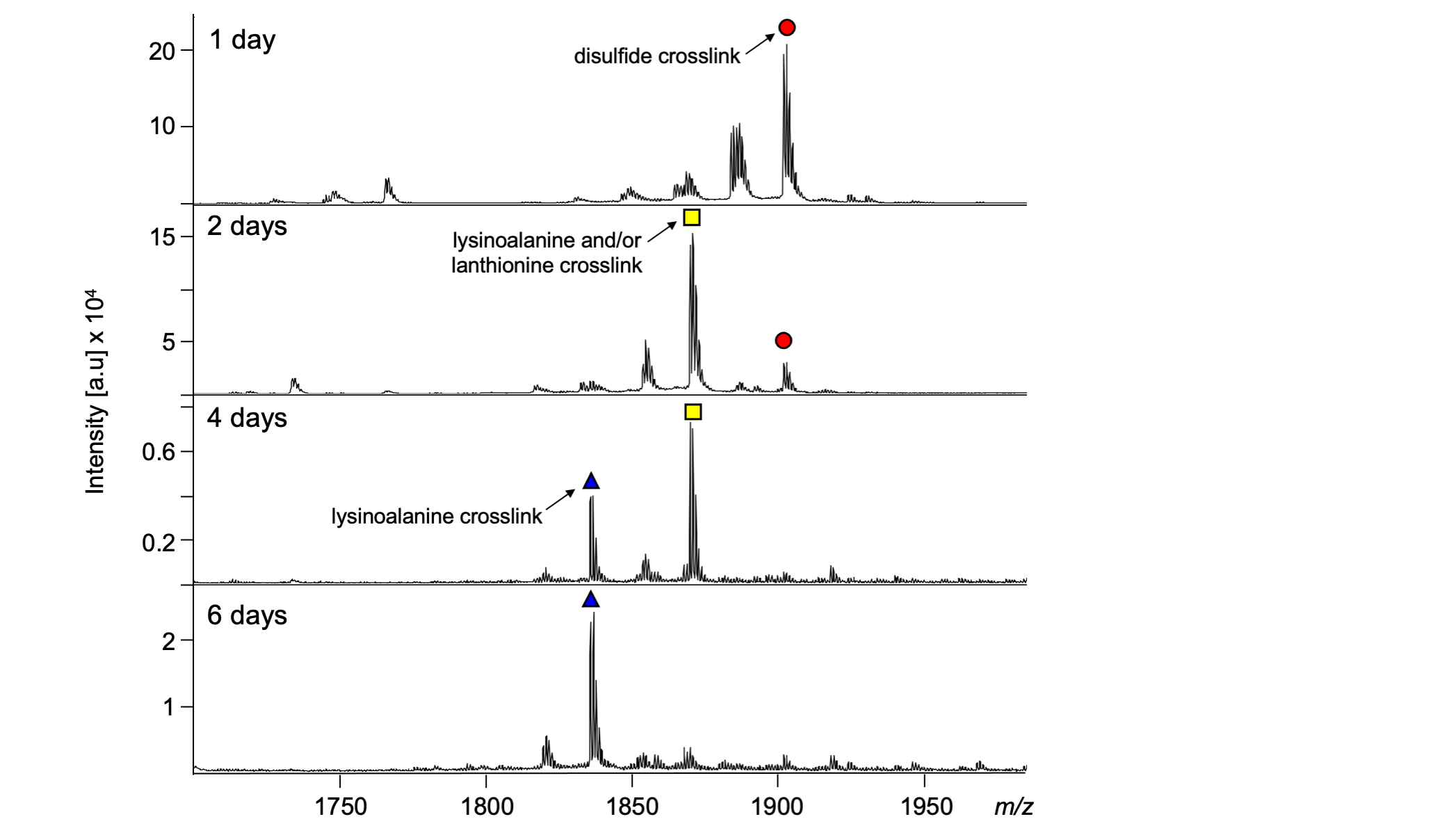
Figure S7. Trend in lysinoalanine and disulfide formation with increasing incubation time.** MALDI MS spectra of LKDECFR heated at 70 °C at pH 12 for either 1, 2, 4, or 6 days, over a 1700–2000 *m/z* range. The disulfide at *m/z* 1901.89 (red circle), a mix of lysinoalanine and/or lanthionine at *m/z* 1869.9 (yellow square), and lysinoalanine at *m/z* 1835.9 (blue triangle) are observed at varying intensities in each spectrum.
